# Multimodal therapy and twenty years of valid management of a patient with chronic hepatitis B in a less developed Western Region in China---case report and review of the literature

**DOI:** 10.1186/s12879-019-3673-4

**Published:** 2019-01-14

**Authors:** Yue-Rong Zhang, De-Ming Wu, Xiong-Chang Liu, Ning Zhou, Chang-Chun Cao, Wu-Hua Liu, Chun-Xia Wang, Hui Wang

**Affiliations:** 1 Department of Infectious diseases, The First People’s Hospital of Lanzhou, No. 1 of Wu jia yuan West Street, Qilihe District, Lanzhou, 730050 Gansu China; 2 Department of Gastroenterology, The First People’s Hospital of Lanzhou, Gansu, 730050 China; 3 Department of Radiology, The First People’s Hospital of Lanzhou, Gansu, 730050 China; 4 Department of general surgery, The First People’s Hospital of Lanzhou, Gansu, 730050 China; 5 Department of laboratory, The First People’s Hospital of Lanzhou, Gansu, 730050 China

**Keywords:** Hepatitis B, Cirrhosis, Multimodal therapy, Disease management

## Abstract

**Background:**

For patients with chronic hepatitis B and cirrhosis in less developed western regions in China, due to constraints of local economic conditions, the choice of treatment measures is often limited. However if patients recieved valid management and effective treatment, they were able to maintain their health and benign prognosis**.**

**Case presentation:**

This study narrates the long-term treatment and careful follow-up of a patient with chronic hepatitis B and cirrhosis in a less developed western region in China, and analyzes the prognosis of the disease and countermeasures.

**Conclusions:**

This would partly reflect the development of antiviral therapy for chronic hepatitis B and multidisciplinary comprehensive treatment for cirrhosis-related complications in remote region with limited resources in the past 20 years.

## Background

The widespread inoculation of hepatitis B vaccine has reduced the infection rate of hepatitis B virus from 9.75%, 20 years ago, to 7.18%, 10 years ago in China [1, 2]. With the development of antiviral drugs and the multidisciplinary comprehensive treatment of cirrhosis, patients can achieve better therapeutic effect and prognosis [3]. However, for patients with chronic hepatitis B and cirrhosis in less developed western regions in China, due to constraints of local economic conditions, the choice of treatment measures is often limited. With the improvement of the overall medical technology level, it has become possible to choose the precise anti-hepatitis B virus therapy and multidisciplinary treatment for cirrhosis-related complications, according to the specific condition of the patients. This allows for the long-term control of the patient’s condition, prevents disease progression, and avoids or delays liver transplantation. Hence, it is a valuable clinical attempt. The diagnosis and treatment of a patient with hepatitis B and cirrhosis is reported, as follows.

## Case presentation

The patient is a male, who was born in October 1968. At present, the patient is a resident of Lanzhou, Gansu Province, and is a worker. He denies any family history of hepatitis B.

This study was conducted in accordance with the declaration of Helsinki and was conducted with approval from the Ethics Committee of The First People’s Hospital of Lanzhou.Written informed consent was obtained from participant.

Primary diagnosis: In June 1996, the patient was hospitalized due to fatigue and yellow staining of the skin and mucosa for 10 days. Serum markers of hepatitis B virus at admission: HBsAg (+), anti-HBe (+), and anti-HBc (+). Liver function: total bilirubin (TBIL), 245.4 μmol/L; alanine aminotransferase (ALT), 324 U/L; aspartate transaminase (AST), 265 U/L. Abdominal ultrasonography results: liver echo enhancement and mild splenomegaly. Clinical diagnosis: active hepatitis B. After liver-protecting treatment, the condition of patient improved, and the patient was discharged.

In March 1998, the patient developed fatigue and yellow staining of the skin and mucosa again for unknown reasons. Furthermore, and the patient had elevated bilirubin and transaminases levels, and the result of the HBVDNA dot-blot hybridization was (+). The patient was given 30 μg of INFa-2b (manufactured in China), *im*, *qod*, and the course of treatment was 24 weeks. In April 2000, the patient had fatigue and a deepening urine color. TBIL was 268.4 μmol/L, ALT was 678 U/L, and AST was 465 U/L. Alpha-fetal protein (AFP) was > 1000 ng/ml, and HBVDNA was 5.4 × 10^5^ cps/ml (the reagents and instruments were purchased from Shenzhen Piji Bioengineering Co., Ltd.; the normal upper limit was 10^3^ cps/ml). Abdominal ultrasonography results: hepatomegaly and splenomegaly. Biopsy of liver tissue: chronic active hepatitis, G3S4. Clinical diagnosis: (1) chronic active hepatitis; (2) early cirrhosis. Treatment regimen: 100 mg/day of lamivudine for 48 weeks, sequential use of INFa-1b (manufactured in China), 50 μg, *im*, three times a week, for 24 weeks.

Examination results of liver function in July 2002: TBIL, 265.1 μmol/L; ALT, 352 U/L; AST, 233 U/L; HBVDNA, 3.6 × 10^4^ cps/ml. Hepatitis B serum markers: HBsAg (+), anti-HBe (+), and anti-HBc (+). Abdominal ultrasonography results: diffuse lesions of the liver, and splenomegaly. The patient began to orally receive 100 mg/day of lamivudine, and has continued this ever since. After 1 week of medication, the level of HBVDNA decreased below the lower detection limit. Gastroscopy in May 2003: moderate esophageal varices (based on the endoscopic criteria developed by the Japanese Society for Portal Hypertension [4], Lm F2 Cb RC+ Lg-).

**Fig. 1:**
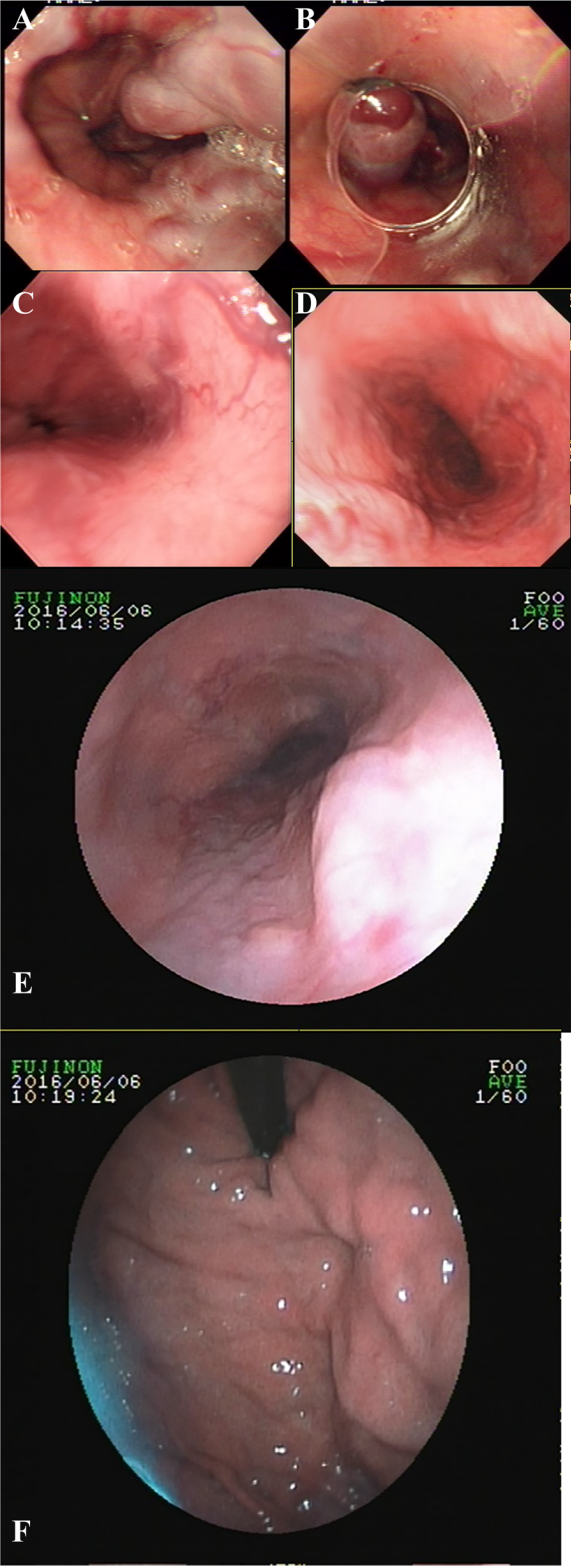
Situations before esophageal variceal ligation and in postoperative follow-ups. **a**. Before treatment in 2004. **b**: During EVL treatment in 2004. **c**. Gastroscopy in 2010. **d**: Gastroscopy in 2012. **e**: Gastroscopy for esophagus in 2016. **f**: Gastroscopy for fundus of the stomach in 2016

The patient was emergently hospitalized for 3 h due to hematemesis in February 2004. Gastroscopy at 3 days after admission: severe esophageal varices (Ls F3 Cb RC+ Lg-). The patient received endoscopic variceal ligation (EVL). Propranolol was applied from the 3rd day after the operation, at 10 mg per time, *bid*. Abdominal ultrasonography results: cirrhosis, splenomegaly, and a small amount of ascites. Clinical diagnosis: decompensated hepatitis and cirrhosis, esophageal varices bleeding, and ascites. Two more EVL operations were performed in August 2004 and September 2005. Thereafter, gastroscopy revealed that the esophageal varices disappeared, and propranolol was discontinued. Thereafter, gastroscopy has been performed every 1–2 years, and obvious varices were not observed. Gastroscopy in 2016: mild esophageal varices (the gastroscopic results are presented in Fig. 1).

**Fig. 2:**
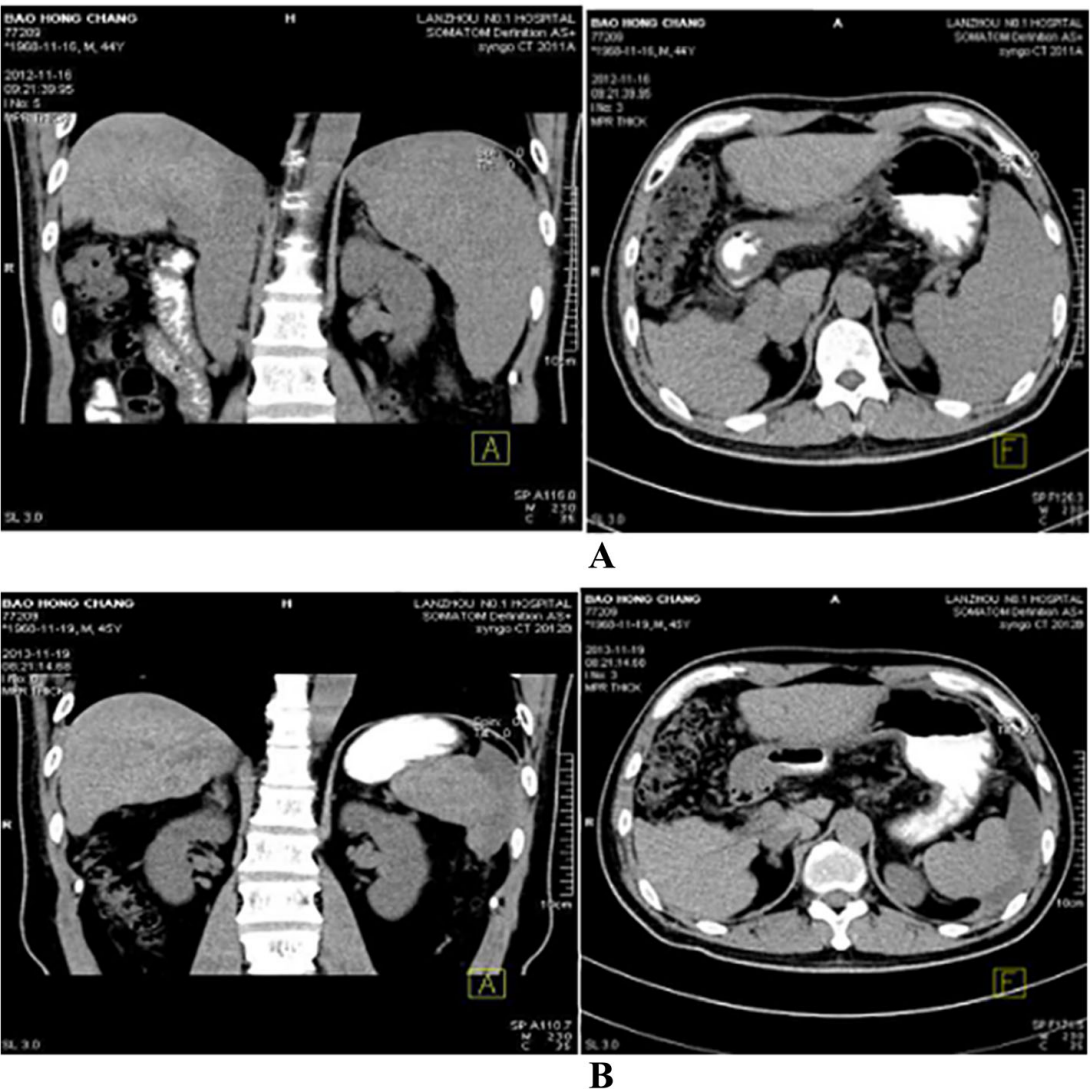
Comparison of upper abdominal CT before PSE and at 48 weeks after PSE. **a**. Upper abdominal non-enhanced CT before PSE and at 1 week after PSE in 2012. **b**. Upper abdominal non-enhanced CT at one year after PSE in 2013

From March 2009 to December 2012, dynamic monitoring revealed that white blood cell (WBC) and platelet (PLT) count gradually decreased. Gastroscopic result: no varices were found at the esophagus and fundus of the stomach. After full communication with the patient, partial splenic artery embolization (PSE) was performed in December 10, 2012. Preoperative routine blood test: WBC, 1.9 × 10^9^/L; NEU, 1.2 × 10^9^/L; PLT, 50 × 10^9^/L. The postoperative monitoring results of the routine blood indexes are presented in Table 1, while the upper abdomen computed tomography (CT) results are presented in Fig. 2.

**Table 1:** Preoperative and postoperative monitoring results of the routine blood indexes of partial splenic artery embolization (A/L)

	WBC(*10^9^/L)	PLT(*10^9^/L)
Preoperative 2012-12-14	1.9	50
2 weeks Postoperation 2012-12-28	6.6	408
3 weeks Postoperation 2013–02	5.8	278
1 year Postoperation 2013–12	3.8	158
2 year Postoperation 2014–11	3.7	126
3 year Postoperation 2015–12	3.6	114
4 year Postoperation 2016–11	3.5	116
5 year Postoperation 2017–12	3.7	113

## Results

Five-year follow-up: The routine blood test, liver function, AFP, HBVDNA, liver elasticity and abdominal ultrasonography were re-checked each half year, and upper abdomen CT was performed every 1–2 years. The results of the related examinations are presented in Tables 1, 2, 3 and 4.

**Table 2:** Hepatitis B serum markers

	HBsAg(COI)	anti- HBs(IU/L)	HBeAg(COI)	HBeAb(COI)	HBcAb(COI)
2012–11	28.1	< 2	0.1	0.011	0.009
2013–11	11.11	< 2	0.076	0.008	0.005
2016–06	5.11	< 2	0.03	0.043	0.003
2017–12	2.36	< 2	0.111	0.084	0.010

**Table 3:** Cobas HBVDNA levels and Determination of lamivudine resistance sites

	HBVDNA manufactured in China	Cobas HBVDNA(IU/ml)	Determination of lamivudine resistance
	< 10^3^cps/ml	–	None
	< 10^3^cps/ml	–	None
2013–11	< 500 IU/ml	91	YMDD tolerance
2014–10	< 500 IU/ml	45	Can’t measure
2016–06	< 500 IU/ml	70	unmeasured
2017–12	< 500 IU/ml	76	unmeasured

**Table 4:** Biochemical criterion and AFP level

	ALT(U/L)	AST(U/L)	TBIL (umol/L)	AFP(ng/ml)	CHE(IU/L)	LSM(kPa)
2013–11	43	50	46.2	< 10	5860	
2014–10	40	53	48.1	< 10	6290	
2016–06	50	54	51	< 10	6354	8.3
2017–12	64	53	40.3	< 10	6512	8.4

Five-year medication: 100 mg of lamivudine, *qd*; 100 mg of silibinin, *tid*.

Present diagnosis: hepatitis B and cirrhosis, compensatory stage

State after EVL for exophageal varices

State after the operation for hypersplenism and partial splenic artery embolization

Live function Child-Pugh grade A

## Discussion and conclusions

1. Medical history characteristics: (1) The patient had an onset of the disease at youth, denied any family history of hepatitis B, and was a hepatitis B patient without mother-to-infant vertical transmission. (2) Since the primarily diagnosis in June 1996, the diagnosis and treatment lasted for 22 years, the patient developed from chronic hepatitis to decompensated cirrhosis, and successively had esophageal varices bleeding, ascites, hypersplenism and other complications [5]. (3) Based on the treatment of nucleoside antiviral drugs, EVL and PSE and other treatments were performed for a number of times, preventing complications such as upper gastrointestinal bleeding, ascites, hepatic encephalopathy, and hypersplenism in the past 10 years, and achieving the goal of “inhibiting viral replication, delaying the progression of the disease, prolonging survival time, and improving quality of life” [6]. This case of multidisciplinary comprehensive treatment achieved a satisfactory curative effect in a less developed region, and is also a relatively successful typical case of the long-term management of chronic diseases.
2. The characteristics of antiviral therapy for hepatitis B: Twenty years ago, interferon was the only drug that could inhibit hepatitis B virus in China [7]. However, the interferon manufactured in China had the characteristics of weak antiviral effect and short half-life. Furthermore, the virus genotype was C-type, and the C-type gene has poor sensitivity to interferon [8]. Therefore, at the initial stage, the interferon therapy performed to patients failed to control the disease, and lead to repeated hepatitis activity. Lamivudine became commercially available in China in 1998. One year later, it entered the Gansu pharmaceutical market. At that time, patients were worried that its “long-term application may induce drug resistance and aggravate the condition” [9]. Hence, the patient received sequential therapy with 48 weeks of lamivudine and 24 weeks of interferon manufactured in China. Nine months after the end of the above-mentioned treatments, the hepatitis became active again. At that time, except for lamivudine, there were no other nucleoside drugs available. Hence, the patient was asked to receive lamivudine treatment again, and has been taking lamivudine for 16 years. The HBVDNA test with a detection reagent manufactured in China was continuously negative. In the last 5 years, the patient underwent Cobas HBVDNA test, and the result fluctuated within 45–91 IU/ml, the drug resistance of lamivudine was detected two times, and YMDD mutation was detected once. In the domestic and foreign guidelines established in recent years, lamivudine was not recommended as the first line anti-hepatitis B virus drug [5, 10, 11]. However, in China, there were still some patients who have been treated with the drug for more than 10 years. The HBVDNA test with a detection reagent manufactured in China was normal, liver function was normal, and the conditions are stable. However, the detection level of Cobas HBVDNA replication remained extremely low. At present, there is no clear recommendation in relevant guidelines at home on whether these patients need to alter antiviral drugs to achieve the goal of undetectable Cobas HBVDNA level. Recent studies abroad have indicated that patients with low levels of HBV DNA have a higher risk of HCC than HBV DNA-negative patients [12], so patients are advised to switch to other first-line nucleoside medicines. to achieve highly Cobas HBV DNA negative.
3. The characteristics of the multidisciplinary comprehensive treatment for cirrhosis-related complications: Cirrhosis-related complications affect multiple organs and systems. Therefore, foreign guidelines recommend that patients with cirrhosis decompensation complicated with multiple complications should be treated with liver transplantation, as soon as possible [13]. In view of the realistic condition that the prevalence of cirrhosis is high and liver resources are insufficient in China, the goal of improving prognosis, improving quality of life and reducing medical burden has been achieved through the implementation of a multidisciplinary comprehensive treatment strategy. In the past 10 years, relevant guidelines and consensus at home and abroad have been constantly updated and refined under the support of evidence-based medicine, leading to the establishment of primary and secondary prevention schemes for esophageal and gastric variceal hemorrhage [14–17]. For patients with acute bleeding,the traditional view that endoscopic therapy plus beta blocker was still the first choice for prevention of variceal rebleeding is updateing [18, 19], bleeding after standard treatment failure, complicated portal vein thrombosis, refractory ascites and non-HVPG response, transjugular intrahepatic portosystemic shunt (TIPS) is more suitable [19–21]. However, there are still the risks of hepatic encephalopathy, stent obstruction and varicose re-bleeding within 2 years after TIPS [22]. When megalosplenia or hypersplenism occurs, the degree of esophageal fundus varicosity and liver function of patients were assessed to determine whether splenectomy combined with disconnection/shunt, PSE, or TIPS would be performed for the patient. PSE is simple in technology, which is safe and has lesser complications. In short, conditions, individual requirements, local medical technology level, economy and other factors affect the choice of treatment measures and curative effect of patients. Under the overall consideration of various factors, a suitable multidisciplinary comprehensive treatment strategy should be chosen for patients, in order to prevent disease progression, and allow more patients to avoid or delay liver transplantation, which can reflect the doctor’s ability of judgment and accurate treatment.

## Abbreviations

AFP: Alpha-fetal protein
ALT: alanine aminotransferase
CT: computed tomography
EFL: endoscopic variceal ligation
PLT: platelet
PSE: partial splenic artery embolization
TIPS: transjugular intrahepatic portosystemic shunt
WBC: white blood cell
